# Case report: recurrent metastatic breast cancer in internal mammary dissection bed discovered at the time of coronary bypass

**DOI:** 10.1186/s13019-019-0980-1

**Published:** 2019-09-05

**Authors:** Gavitt A. Woodard, Hannah Lee, Daffolyn Rachael Fels Elliott, Kirk D. Jones, Jasmine Wong, David M. Jablons, Kai Ihnken

**Affiliations:** 1 Department of Surgery, University of California San Francisco, 500 Parnassus Avenue, Room MUW-424, San Francisco, CA 94143-1724 USA; 2 Department of Pathology, University of California San Francisco, 505 Parnassus Avenue, Room M-545, San Francisco, CA 94143-1724 USA; 3 Department of Surgery, University of California San Francisco, 1600 Divisadero Street, 2nd Floor, Box 1710, San Francisco, CA 94115 USA; 4 Department of Surgery, University of California San Francisco, 500 Parnassus Avenue, Room MUW-405, San Francisco, CA 94143-1724 USA

**Keywords:** Recurrent breast cancer, Chest wall radiation, Left internal mammary artery dissection, Internal mammary lymph node, Hormonal therapy tumor clonal selection

## Abstract

**Introduction:**

Many patients who undergo coronary artery bypass surgery have a prior history of cancer and potentially chest radiation which is a known risk factor for coronary atherosclerosis. Prior radiation increases fibrosis and can make the dissection of the left internal mammary artery (LIMA) more challenging.

**Case report:**

A 72-year-old woman with a history of stage IIA pT2N0M0 left breast intraductal carcinoma treated with lumpectomy, adjuvant chemotherapy and radiation therapy 11 years prior presented to the emergency room with a non-ST elevation myocardial infarction and was taken for cardiac catheterization followed by three-vessel coronary artery bypass grafting. The LIMA was found to be encased in scar tissue and was deemed unsuitable as a conduit, and a saphenous vein graft was bypassed to the left anterior descending artery in its place. Pathologic review of the LIMA showed nests of tumor cells infiltrating within dense fibrous tissue with areas of necrosis and calcifications consistent with recurrent breast cancer. Interestingly the patients original breast cancer was positive for estrogen receptors (ER) and progesterone receptors (PR) ER and PR and negative for HER2 and she had therefore been treated with 5 years of hormonal therapy. The recurrent cancer found in the LIMA dissection bed at the time of bypass surgery was ER, PR, and HER2 negative, suggesting hormonal therapy driven clonal selection of these metastatic tumor cells.

**Discussion and conclusions:**

Scarring in the LIMA dissection bed in patients with a history of cancer and prior chest radiation should be carefully evaluated for the possibility of recurrent cancer. The gross appearance of tissue can be misleading and sending a biopsy for a formal frozen section histologic evaluation should be considered if there is any question of recurrent malignancy.

## Introduction

Breast and chest wall radiotherapy for the treatment of breast cancer, in particular for left breast cancer, is known to expose the heart to incidental ionizing radiation. This exposure increases the risk of development of coronary artery disease by 30% and cardiac mortality by 40% [1]. The risk is greatest in female smokers who have a 1% risk of cardiac mortality following radiotherapy versus nonsmokers 0.3% risk. This increased cardiac mortality has led some authors to suggest that radiotherapy for breast cancer be tailored based on a patient’s individual cardiac risk factors [2]. Prior chest radiation therapy increases tissue scarring and makes the dissection of the left internal mammary artery (LIMA) conduit for coronary bypass more challenging. There are no reported cases of recurrent breast cancer arising in the LIMA dissection bed in the literature.

## Case report

A 72-year-old woman with a history of hyperlipidemia, pre-diabetes and stage IIA pT2N0M0 left breast intraductal carcinoma treated with lumpectomy, adjuvant chemotherapy and radiation therapy 11 years prior presented to the emergency room with acute on chronic chest pain. Upon presentation she reported a history of several months of intermittent substernal chest pain with exertion that was relieved with rest. In 2003 the patient had undergone a screening coronary calcification test scoring 1744, at the 99th percentile for age and gender. Coronary CT angiography at that time demonstrated a < 30% lesion in the proximal right coronary artery, minimal irregularities in the circumflex and obtuse marginal arteries and sequential 30% lesions in the mid left anterior descending artery. One month prior to presentation she underwent a Lexiscan stress electrocardiogram (ECG) study and single-photon emission computerized tomography (SPECT) myocardial perfusion study which appeared grossly normal but demonstrated decreased radiotracer uptake at the apex consistent with apical thinning artifact given normal wall motion and left ventricular ejection fraction > 65%. Lexiscan stress ECG was normal.

At the time of admission her troponin was 0.05 ng/ml and she was treated with sublingual nitroglycerin, which relieved her chest pain. She was started on aspirin, pravastatin, metoprolol, heparin infusion, and went to catheterization laboratory for coronary angiogram within 24 h. Angiogram demonstrated severe 80% stenosis of the distal left main coronary artery, moderate to severe stenosis of the proximal left anterior descending, and tandem 95% proximal-mid lesions of the right coronary artery, with left-to-right collaterals to the distal right coronary circulation, and she was therefore referred to cardiac surgery for urgent three-vessel coronary artery bypass surgery.

She went to the operating room two days later. During takedown of the LIMA a six cm length of dense adhesions was noted and presumed to be related to her prior history of radiation for breast cancer. The LIMA proved to be encased in scar tissue and was deemed unsuitable as a conduit, and a saphenous vein graft was bypassed to the left anterior descending artery in its place. Due to the dense nature of the scar tissue on the mammary wall a specimen was sent to pathology.

**Fig. 1:**
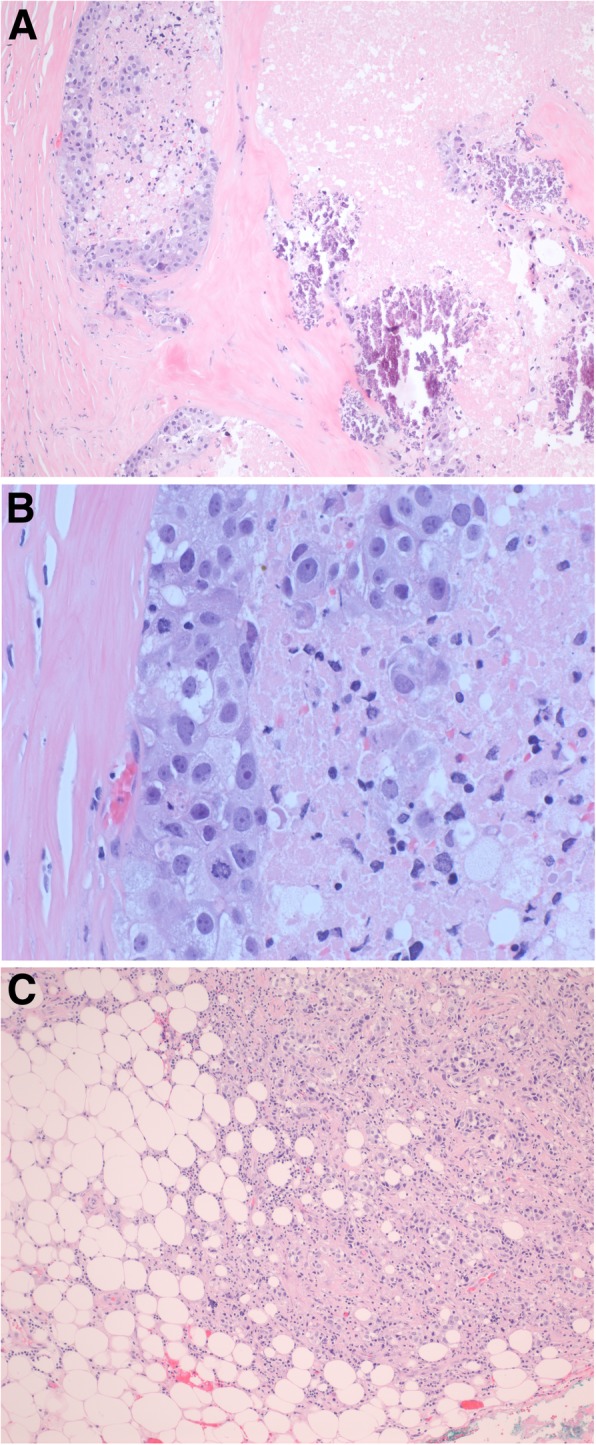
Histologic features of the left chest soft tissue mass. **a** Nests of tumor cells demonstrating necrosis and calcifications within a background of dense fibrous tissue. Magnification 100X. **b** Tumor cells have abundant clear to eosinophilic cytoplasm, pleomorphic nuclei, prominent nucleoli, and brisk mitotic activity. Magnification 400X. **c** Tumor cells appear to be invading into adjacent fat. Magnification 100X. Hematoxylin and eosin (H&E) stains

**Fig. 2:**
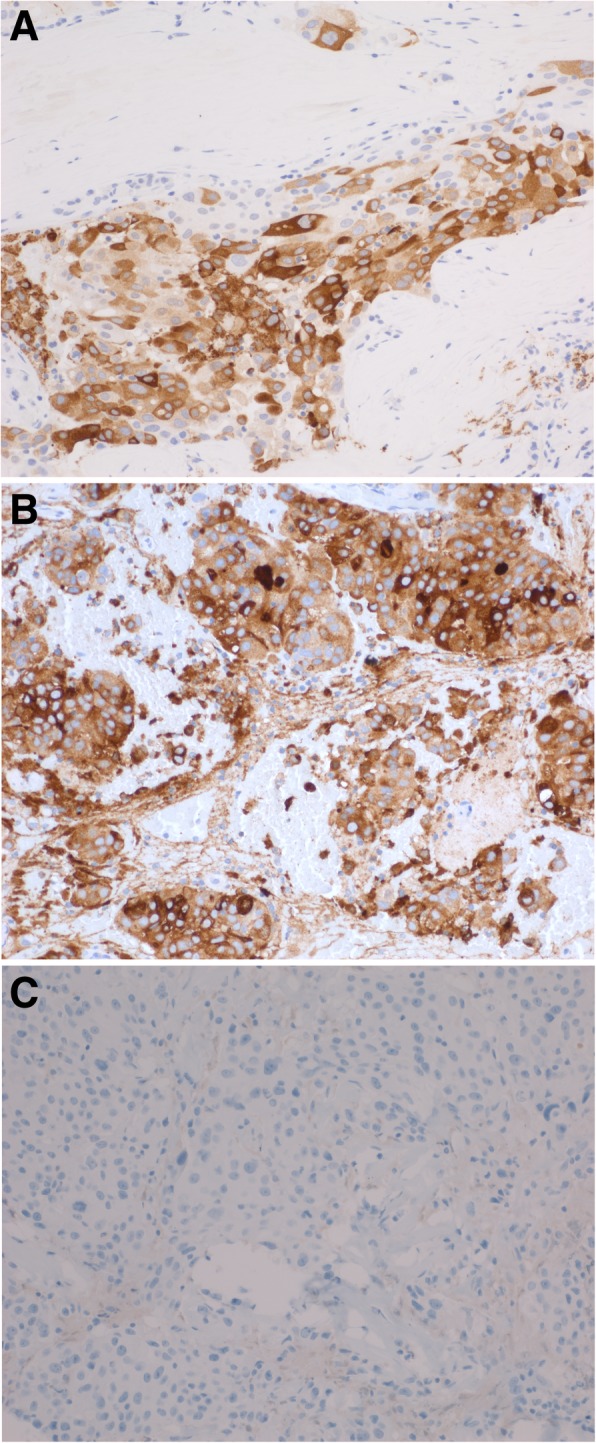
Immunohistochemical profile compatible with breast origin. Tumor cells show positive cytoplasmic staining for **a** mammaglobin and **b** GCDFP-15, immunohistochemical markers consistent with breast primary origin. **c** Immunohistochemical staining for estrogen receptor is negative. Magnification 200X

Hematoxylin and eosin-stained histologic sections of the surgical specimen, designated left chest soft tissue mass, showed nests of tumor cells infiltrating within dense fibrous tissue with areas of necrosis and calcifications (Fig. 1a). The tumor cells had clear to eosinophilic cytoplasm, pleomorphic nuclei, prominent nucleoli and brisk mitotic activity (Fig. 1b). The tumor cells extended into adjacent adipose tissue (Fig. 1c) and were present at the resection margin. Immunohistochemical stains showed positive cytoplasmic staining for mammaglobin and gross cystic disease fluid protein 15 (GCDFP-15) (Fig. 2a-b) but negative staining for thyroid transcription factor 1 (TTF-1), Wilm’s tumor protein 1 (WT-1), calretinin, and GATA3. Immunohistochemistry for estrogen receptors (ER) (Fig. 2c) and progesterone receptors (PR) was negative and human epidermal growth factor receptor 2 (HER2) was equivocal (staining intensity 2+, scale 0–3+). Confirmatory fluorescence in situ hybridization (FISH) testing showed no amplification of HER2. Overall, the morphology and immunophenotype was most consistent with metastatic/recurrent breast carcinoma.

By report, the patient’s prior left breast cancer was a two cm invasive ductal carcinoma positive for ER and PR and negative for HER2 by immunohistochemistry. The Ki67 proliferation index was estimated at 40%. As her surgical specimen was node negative without lymphovascular invasion she was treated with lumpectomy, followed by four cycles of docetaxol and cyclophosphamide, radiation therapy and anastrazole for 5 years.

## Discussion

This case is an example of a patient with a remote cancer history and prior chest radiation with a challenging LIMA dissection due to unrecognized recurrent malignancy. The gross appearance of her recurrent tumor was initially suspected to be scarring from radiation fibrosis and only a formal histologic evaluation yielded the diagnosis of malignancy. The recurrent breast cancer in this patient did not appear confined to a lymph node, but may have arisen from undetected metastases to the internal mammary lymph nodes (IMN) that run along the LIMA. IMNs are a major pathway of lymphatic drainage for the breast and IMN involvement has been reported in approximately 20% of breast cancers at the time of presentation [3]. Routine biopsy and pathologic evaluation of IMNs is generally not performed due to their deep location. Risk of IMN metastasis correlates with axillary lymph node involvement and increases with the number of involved axillary lymph nodes; however, involvement of IMN alone is not an independent predictor of overall survival or disease free survival [3, 4].

Also notable in this case is the down-regulation of hormone receptor expression in comparison to the original tumor. Discordance in hormone receptor and/or HER2 status has been reported in locoregional recurrences and distant metastases in up to 50% of cases [5–7]. In particular, the down-regulation of hormone receptors is 2–4 times more likely to occur after hormonal therapy with reported loss of ER in 10–30% and PR in 20–50% of cases [5]. Loss of ER and/or PR in recurrent breast cancer is considered a poor prognostic sign, and has been shown to correlate with increased risk of death (hazard ratio (HR) 3.62; 95% confidence interval (CI) 1.65–7.94) and (HR 2.34; 95% CI 1.01–5.47) compared with patients with stable ER or PR positive tumors [8, 9]. This increased risk of mortality may be driven by clonal selection for more aggressive, chemotherapy refractory tumor cell populations.

## Conclusions

In summary, scarring on the chest wall or in the LIMA dissection bed is not uncommon in patients with a history of chest radiation who require a coronary bypass operation. However, as this case demonstrates, recurrent cancer cannot be ruled out based on gross appearance alone and we recommend surgeons have a low threshold for sending specimens of what may appear to be radiation scar for formal histologic evaluation.

## Data Availability

Not applicable.

## Abbreviations

CI: Confidence interval
ECG: Electrocardiogram
ER: Estrogen receptor
FISH: Fluorescence in situ hybridization
GCDFP-15: Gross cystic disease fluid protein 15
HER2: Human epidermal growth factor receptor 2
HR: Hazard ratio
IMN: Internal mammary lymph nodes
LIMA: Left internal mammary artery
PR: Progesterone receptors
SPECT: Single-photon emission computerized tomography
TTF-1: Thyroid transcription factor 1
WT-1: Wilm’s tumor protein 1
